# The joint action on healthy life years (JA: EHLEIS)

**DOI:** 10.1186/0778-7367-71-2

**Published:** 2013-02-04

**Authors:** Jean-Marie Robine, Emmanuelle Cambois, Wilma Nusselder, Bernard Jeune, Herman Van Oyen, Carol Jagger

**Affiliations:** 1 French National Institute of Health and Medical Research, INSERM U988 & U710, Paris & Montpellier, France; 2 French National Institute for Demographic Studies, INED, Paris, France; 3 Erasmus Medical Center, University Medical Center Rotterdam, Rotterdam, Netherlands; 4 University of Southern Denmark, Odense, Denmark; 5 Scientific Institute of Public Health, Brussels, Belgium; 6 Newcastle University, Newcastle upon Tyne, United Kingdom

**Keywords:** Health status indicators, Gender, Socioeconomic status, Public health, Health expectancy, Healthy life years, EU

## Abstract

**Background:**

Life expectancy has been increasing during the last century within the European Union (EU). To measure progress in population health it is no longer sufficient to focus on the duration of life but quality of life should be considered. Healthy Life Years (HLY) allow estimating the quality of the remaining years that a person is expected to live, in terms of being free of long-standing activity limitation. The Joint Action on Healthy Life Years (JA: EHLEIS) is a joint action of European Member States (MS) and the European Union aiming at analysing trends, patterns and differences in HLY, as well as in other Summary Measures of Population Health (SMPH) indicators, across the European member states.

**Methods:**

The JA: EHLEIS consolidates existing information on life and health expectancy by maximising the European comparability; by analysing trends in HLY within the EU; by analysing the evolution of the differences in HLY between Member States; and by identifying both macro-level as micro-level determinants of the inequalities in HLY. The JA: EHLEIS works in collaboration with the USA, Japan and OECD on the development of new SMPHs to be used globally. To strengthen the utility of the HLY for policy-making, annual meetings with policy-makers are planned.

**Results:**

The information system allows the estimation of a set of health indicators (morbidity and disability prevalence, life and health expectancies) for Europe, Member States and shortly their regional levels. An annual country report on HLY in the national languages is available. The JA: EHLEIS is developing statistical attribution and decomposition tools which will be helpful to determine the impact of specific diseases, life styles or other determinants on differences in HLY. Through a set of international workshops the JA: EHLEIS aims to develop a blueprint for an international harmonized Summary Measure of Population Health.

**Conclusion:**

The JA: EHLEIS objectives are to monitor progress towards the headline target of the Europe 2020 strategy of increasing HLY by 2 years by 2020 and to support policy development by identifying the main determinants of active and healthy ageing in Europe.

## Background

Life expectancy at birth has steadily increased during the last decade in the EU27, by more than 3 years for men and 2 years for women, leading to accelerated population ageing. However gaps among Member States of the European Union (EU) have remained large, 13 years in both 1997 and 2010 for men, although for women gaps have slightly reduced from more than 9 years in 1997 to 8 years in 2010. Additionally the gender gap in life expectancy at birth remains high at 6 years in 2010 although it also has reduced over time. When the quality of the years lived is taken into account through health expectancies such as life expectancy in good perceived health, life expectancy without chronic morbidity, or life expectancy without disability, even larger gaps are seen among European countries. For instance, life expectancy without long-standing activity limitations or Healthy Life Years (HLY) show gaps in both men and women reaching close to 20 years in 2010 among Member States [1].

The HLY is technically a Summary Measure of Population Health and part of the family of disability-free life expectancy indicators which measure the number of remaining years that a person of a certain age is expected to live without disability. A Summary Measure of Population Health brings together data on both the length and the quality of life [2] and are considered important population health outcome measures [3]. In the HLY indicator, disability is measured through the global activity limitation indicator (GALI) which is a single survey question capturing long-standing limitations in usual activities [4,5]. In 2005 it was included as the only Lisbon Structural Indicator on health with the main purpose to monitor health trends and health gaps in Europe [6]. The utility and relevance of HLY for public health depends on a number of issues being resolved, including: maximising the pan-European comparability of the measure of long-standing limitations; decomposition of the gaps in HLY by origin (mortality or disability), or by cause of morbidity and age group responsible; and the ability to assess the impact of changes in health determinants and health interventions on HLY. For instance, there is some evidence suggesting that the effect of health determinants such as smoking decreases the total life expectancy and decreases both the years without and with disability, while other determinants such as obesity decrease the years without disability but increases the years with disability [7]. At a national and EU level, the utility of HLY also depends on whether it can be computed by population sub-groups (i.e. socio-economic groups) and by sub-national units.

As measures of population health, HLY and health expectancies in general have several strengths. Health expectancy indicators are more intuitive and understandable for policy makers and the public at large compared to mortality rates or morbidity incidence or prevalence rates. Health expectancies are typically estimated based on cross-sectional survey data [8] and guidelines for calculation have been made freely available [9]. However the use of varying definitions of health, morbidity and disability has hampered the international comparability of health expectancy indicators and for their ability to monitor time trends in population health. The international research network REVES (Réseau Espérance de Vie en Santé – International Network on Health Expectancies) was set up in 1989 to tackle these issues and to work on definitions, measurements and comparison of disability globally [10]. Following the Maastricht Treaty in 1993 the EU initiated horizontal actions to develop health monitoring and health indicators [11] including health expectancies which were the focus of two EURO-REVES projects. A major deliverable of the Euro-REVES projects was the development of a coherent set of instruments to monitor health across Europe [12] and the successful introduction of these common instruments into several European surveys such as the European Health Interview Survey (EHIS) and the European Survey on Income and Living Conditions (EU-SILC) enabled for the first time estimation of HLY in all EU Member States in a harmonised way.

The next step in this process was to structure and harmonize the calculation, analysis and interpretation of data collected through those surveys. This goal was achieved by the European Health Expectancy Monitoring Unit (EHEMU) and the European Health and Life Expectancy Information System (EHLEIS) project. EHEMU established an information system which facilitates the calculation of life and health expectancies in the EU and its Member States. This information system provides a central facility for the co-ordinated analysis and synthesis of life and health expectancies and provides evidence of inequalities between Member States, highlighting potential targets for public health strategies both nationally and at a pan-European level. EHLEIS extended the EHEMU activities by more in-depth analysis of trends and gender gaps in health expectancies [13,14] and of how the similarity or differences between groups of Member States are correlated with macro level factors covering broad areas of wealth and expenditure (GDP, poverty risk, inequality of income distribution, expenditure on elderly care, labour force participation (employment rate of older at age 55–64 years, long-term unemployment rate, mean exit age from labour force), and level of education (life-long learning, low education attainment)) [15,16]. Additionally the EHLEIS project provided training workshops to ensure best practice and a deeper understanding of health expectancy indicators, and, through regular communication, enabled policy makers, public health officials and researchers to exchange information on the utility of the health expectancy indicators and how they are incorporated in government strategies in the EU Member States [17]. From 2011 onwards projects on health expectancies are conducted within the framework of a Joint Action on Healthy Life Years (JA: EHLEIS) among the European Commission and the Member States.

### The joint action on healthy life years

Within the programme of the Community action in the field of health [18], JA: EHLEIS addresses the topic “*Maintain and continue developing a European Health and Life Expectancy Information System*, *in order to improve and harmonise calculations for the development of the Healthy Life Years* – *HLY* – *structural indicator*”. Accordingly the JA: EHLEIS

(i) consolidates the existing information system (EHLEIS) through promoting further European comparability, annual HLY calculations and dissemination (online information system, annual country reports [1]) and updating of trends and gap analysis at the EU level, as well as the role of macro-level factors in explaining these gaps,

(ii) further extends EHLEIS through analyses of micro-level health determinants using the new European Health Interview Survey (EHIS) and analyses of HLY gaps between socio-economic groups,

(iii) develops an alternative Summary Measure of Population Health that can also serve as a common international measure beyond the EU,

(iv) Integrates the Task Force on Health Expectancies [19] into an annual meeting to further engage Member States and promote HLY use in policy-making within Member States.

In particular, in the trend and gap analyses the JA: EHLEIS utilises a range of health expectancies, including self-rated health, morbidity and disability. The JA also develops a study design for computing maximally comparable health expectancies by socio-economic status for the Member States that already have mortality data by socio-economic position. Finally, in collaboration with the main OECD countries, especially the United States of America (USA) and Japan, the JA: EHLEIS will provide a blue print for an improved new internationally comparable Summary Measure of Population Health, thus upgrading the current European HLY by a global indicator. A Summary Measure of Population Health that is comparable to the USA and Japan indicators is a necessary condition to achieve a grade “A” for European Structural Indicators. The international discussion will not focus on the health expectancy indicator as such but rather on the essential of how to measure the health or quality of life related information necessary to calculate the health expectancy.

### Target groups

The JA: EHLEIS targets Member States, health and non-health policy makers at the regional, national and European level, health professionals and researchers, media, general public and Non Governmental Organizations (NGO). Increasing involvement of Member States in health monitoring is one of the main priorities of JA: EHLEIS as well as promoting their wider engagement in using HLY in policy-making. The annual public JA: EHLEIS meeting plays a central role in addressing these priorities. In addition, the work published by the EHLEIS group in *The Lancet* in 2008 is a good example of HLY use in non-health policies [15]. Scientific material will be provided to the health professional and research communities through: databases, technical reports and scientific publications, adding to the papers already published by EHLEIS on the validity of the GALI-instrument [4,5] and on trends, socio-economic or gender inequalities [13,14,16,20,21]. Additional, special attention will be paid to provide easier access to HLY information for the media, general public and NGOs. EHLEIS (2007–2010) previously produced easy-to-read material, such as an Interpreting Guide to HE, as well as an annual 4-page Country report [1], a general public website [22] and Wikipedia pages [23]. The JA: EHLEIS will continue to develop similar readily accessible material.

## Methods

A first major aim of the Joint Action is to maintain and continue developing the European Health and Life Expectancy Information System (EHLEIS), by providing online calculations of various health indicators (prevalence, LE, HEs including HLY) with confidence intervals, that are comparable among EU countries, and with improved dissemination, understanding, ease of access and public health relevance. The methods and means to meet this aim include: computational and web methodologies for the EHLEIS database; statistical and demographic analyses for the substantive results; linguistic methods for the translation/provision of the easy-to-use web facilities in multiple European languages and translation of the Country reports into national languages; and methodologies to address the conceptual basis of the HLY indicator and its greater comparability with Summary Measures of Population Health in the USA and Japan. The JA: EHLEIS further develops the EHLEIS system to allow rapid access to up-to-date data on health expectancies (and the underlying health/disability prevalence and mortality data) from a variety of European databases (SILC, Survey of Health, Ageing, and Retirement in Europe (SHARE), EHIS). The computational and web expertise to undertake this key foundation of the Joint Action come from Montpellier RIO Imaging [24]. Analysis and interpretation of data and computed indicators are other important aims of the Joint Action. Two work packages are totally devoted to these activities using a wide range of statistical methods at the European, national, sub-national and individual level to perform macro, micro or multi-level analyses, respectively, with the aim to explain trends over time in life and health expectancies (including HLY) and gaps between Member States, genders and socio-economic groups using amongst others health expectancy decomposition tools [25]. Specific analyses will highlight factors explaining limitations in usual activities and the origins of the gaps. “What if” scenario analyses may help quantification of the impact on HLY of certain health behaviour (for instance smoking) or public health interventions. The necessary statistical expertise is provided by the EHLEIS associated partners (Belgium Scientific Public Health Institute, French national institutes for demographic studies (INED) and for health and medical research (INSERM), Erasmus MC, and Newcastle University). Preparation for the next Summary Measure of Population Health, the successor of the HLY, is the second major aim of the Joint Action. To meet this objective the JA: EHLEIS organizes a series of seminars with experts from the USA, Japan and other OECD countries to agree on the conceptual basis for the new Summary Measure of Population Health. The whole Action is coordinated by the French National Institute of Health and Medical Research INSERM.

## Results and discussion

The main outcomes of the Joint Action will comprise:

(i) An Information System allowing online calculation of health indicators (prevalence, life and health expectancies (including HLY)), combining population and mortality data with health information coming from European (SILC, SHARE, EHIS) and/or national surveys. All HLY-related websites will be organized in a new website [1];

(ii) Country reports on HE: Part of a series, each annual report presents life expectancy and HLY for the country of interest and for the overall EU27 using the SILC question on long term activity limitation (GALI) included in the SILC survey since 2004 or 2005. The reports will be translated in the national languages and will be disseminated to national institutions;

(iii) Proceedings of the JA: EHLEIS annual meetings: The annual meetings will consist of one half-day open to the public and media (back to back with the Enlarged Steering Committee). The main aim of the meeting is to encourage Member States to use health expectancies, including HLY, in their health, social and employment policies;

(iv) Presentation of the new HLY values and latest trends in Europe and press communiqués;

(v) Statistical tools - New version of attribution and decomposition tools, distributed with updated manuals through the website [1] and development of impact assessment tool for HLY;

(vi) Technical reports and scientific analyses exploring geographical variations in HLY within Europe, trends in HEs in Europe, social differentials in HLY across Member States and calibration of the Global Activity Limitation Indicator (GALI) with EHIS data;

(vii) A blueprint for a new international Summary Measure of Population Health.

In addition the Joint Action plans to present its work at major European Conferences such as the European Health Forum in Gastein [26] and the EUPHA Conferences [27].

## Conclusion

The JA: EHLEIS contributes directly to two of the three objectives of the Second Programme of Community Action in the Field of Health 2008–2013: to promote health and to generate and disseminate health information and knowledge [18]. The JA: EHLEIS also contributes to the Europe 2020 strategy and its innovation partnership on active and healthy ageing with the target of increasing by 2 years the average number of healthy life years by 2020 [28]. The Joint Action not only offers the means to monitor this headline target, it further contributes towards identifying and understanding of the main determinants of healthy life in Europe, thus offering new avenues for policies targeting the expected increase in HLY. As such the JA: EHLEIS is an important contributor to the 2012 European Year for Active Ageing and Solidarity between Generations [29].

## Competing interests

The authors declare that they have no competing interests.

## Authors’ contributions

JMR and CJ drafted the manuscript. All authors commented the draft versions. All authors read and approved the final manuscript.

## Authors’ information

The JA: EHLEIS team: http://www.eurohex.eu/index.php?option=aboutehemu#team.
